# Multiple hybridization events between *Drosophila simulans* and *Drosophila mauritiana* are supported by mtDNA introgression

**DOI:** 10.1111/j.1365-294X.2010.04838.x

**Published:** 2010-11

**Authors:** Maria D S Nunes, Pablo Orozco-Ter Wengel, Michaela Kreissl, Christian Schlötterer

**Affiliations:** Institut für Populationsgenetik, Veterinärmedizinische Universität WienVienna, Austria

**Keywords:** *Drosophila mauritiana*, *Drosophila simulans*, Hybridization, introgression, maIII, mtDNA, siII

## Abstract

The study of speciation has advanced considerably in the last decades because of the increased application of molecular tools. In particular, the quantification of gene flow between recently diverged species could be addressed. *Drosophila simulans* and *Drosophila mauritiana* diverged, probably allopatrically, from a common ancestor approximately 250 000 years ago. However, these species share one mitochondrial DNA (mtDNA) haplotype indicative of a recent episode of introgression. To study the extent of gene flow between these species, we took advantage of a large sample of *D. mauritiana* and employed a range of different markers, i.e. nuclear and mitochondrial sequences, and microsatellites. This allowed us to detect two new mtDNA haplotypes (MAU3 and MAU4). These haplotypes diverged quite recently from haplotypes of the siII group present in cosmopolitan populations of *D. simulans*. The mean divergence time of the most diverged haplotype (MAU4) is approximately 127 000 years, which is more than 100 000 years before the assumed speciation time. Interestingly, we also found some evidence for gene flow at the nuclear level because an excess of putatively neutral loci shows significantly reduced differentiation between *D. simulans* and *D. mauritiana*. Our results suggest that these species are exchanging genes more frequently than previously thought.

## Introduction

What defines a species? It is clear from the numerous definitions used that there is no universal concept with which all biologists would agree (Coyne & Orr 2004). This difficulty arises directly from the continuous nature of the process that can ultimately lead to speciation (Darwin 1859; Coyne & Orr 2004; Mallet 2006). More recently, however, it has become increasingly accepted that species can exchange genes and still maintain their species integrity. An interesting and still unanswered question, therefore, is how much gene flow can be tolerated without losing species identity?

*Drosophila simulans* and *Drosophila mauritiana* are an ideal model system to address this question. Their separation occurred probably 250 000 years ago (Kliman *et al*. 2000; McDermott & Kliman 2008). Since then, these species have accumulated several morphological and behavioural differences (e.g. Cowling & Burnet 1981; Robertson 1983; Cobb *et al*. 1988; Lachaise *et al*. 1988; Orgogozo & Stern 2009), as well as numerous hybrid incompatibilities (Coyne & Charlesworth 1986; True *et al*. 1996b; Ting *et al*. 2000; Tao *et al*. 2003a,b; Cattani & Presgraves 2009). Some of these differences might contribute to the maintenance of their reproductive isolation. However, laboratory crosses show that isolation is incomplete because hybrid females are fertile and can have fertile offspring when backcrossed to males of either parental species. Furthermore, introgression of mtDNA has been detected for these species, indicating that hybridization occurred in nature (Solignac & Monnerot 1986; Aubert & Solignac 1990; Ballard 2000a,b;). Multilocus polymorphism data have been used to investigate whether there has been recent gene flow from *D. simulans* into *D. mauritiana* (Hey & Kliman 1993; Kliman *et al*. 2000; Noor & Kliman 2003; McDermott & Kliman 2008). However, although little evidence was found supporting gene flow, the power of these studies may have been limited because either only a small number of loci or a small number of individuals were analysed.

In this study, we take advantage of a large sample of *D. mauritiana* and of different types of markers (i.e. nuclear and mitochondrial sequences, and microsatellites) to investigate the extent of hybridization between *D. mauritiana* and *D. simulans*.

Our results show that at least two, and possibly three, independent hybridization events that led to the introgression of mtDNA from one species into the other occurred in the recent history of these species. In addition, we found an excess of nuclear loci showing significantly low differentiation, which possibly indicates that they are located in regions of the nuclear genome particularly permeable to gene flow between species.

## Materials and methods

### Population samples and data sets

In this study, we analysed 125 *Drosophila mauritiana* isofemale lines representing fly collections over a 28- year period as well as multiple sampling localities from the same year (Table S1). Three of these collections have been maintained in the laboratory as isofemale lines (MAU, MS and RED). The MAU samples were acquired many years before the closure of the Bowling Green stock centre. Several lines exhibited visible markers resulting from spontaneous mutations in wild-type stocks. The exact origin and degree of relatedness of the marker stocks are not known.

To detect the introgression, we sequenced fragments of four mitochondrial genes (*mt:CoI*, *mt:ND4*, *mt:ATPase6* and *mt:ND5*) and three nuclear loci (*pcl*, *fog* and *mav*) from the DNA of single individual females from each of the samples. We also characterized their genotypes at 25 microsatellite loci (eight individuals had to be removed from the data set because of incomplete genotypes).

*pcl* and *fog* are located on the X chromosome, at the telomere (cytological bands 1B2-1B2 of *Drosophila melanogaster*) and centromere (20D2-20E1 of *D. melanogaster*), respectively. The tips of X-chromosome exhibit low rates of recombination in *Drosophila simulans* and *D. melanogaster*, but normal recombination rates in *D. mauritiana* (True *et al*. 1996a). It should be noted, however, that these two genes lie downstream and upstream of the markers that delimit the recombination map. Although there is an overrepresentation of hybrid male sterility factors on the X chromosome (True *et al*. 1996b), none mapped to the tips of the chromosome. Nevertheless, after we completed our data collection, a locus involved in hybrid inviability (*hlx*) was mapped to the centromeric heterochromatin of the X chromosome (Cattani & Presgraves 2009). Hence, we do not know to what extent the pattern of variability of *fog* is affected by this. *mav* is located in the lowly recombining 4th chromosome (102C-102C of *D. melanogaster*). This locus was chosen because the whole 4th chromosome was shown to lack hybrid sterility factors in crosses between *D. mauritiana* and *D. simulans* (Coyne & Berry 1994).

Mau24, Mau17, RB11 and RED25 were sequenced for fragments of two additional mtDNA genes, *mt:ND2* and *mt:Cyt-b*. Primer sequences, amplification product length and annealing temperature are listed in Table S2 for all loci.

Sequence data for the first four mtDNA fragments and the three nuclear loci were obtained from individuals belonging to seven *D. simulans* populations (Table S1).

For the microsatellite-based comparisons between *D. mauritiana* and *D. simulans*, we took advantage of a set of 15 *D. simulans* individuals previously genotyped in our laboratory. These individuals are samples from Asia (China), Europe (Portugal and Italy) and South America (Brazil). Two previously genotyped Zimbabwean populations of *D. melanogaster* (11 and 21 isofemale inbred lines from Sengwa and Victoria Falls respectively) were used for the analyses, where a comparison to an outgroup was required (Table S1). The raw data for the microsatellites are available in Dryad (doi: 10.5061/dryad.1731).

### Screening of Wolbachia

To test our samples for the presence of *Wolbachia*, we used primers that are conserved in several *Wolbachia* sequences available in GenBank and which belong to strains that were isolated from various *Drosophila* species (Table S2).

### DNA extraction, amplification and sequencing

DNA was isolated from individual females of each strain by a high-salt extraction method (Miller *et al*. 1988). Standard amplification conditions were 35 cycles of denaturation at 94 °C for 50 s, primer annealing temperature as indicated in Table S2 and primer extension at 72 °C for 50 s. PCR products were purified using 96-well plates (Millipore) according to the supplier's protocol. All PCR products were sequenced in both directions with primers used for the fragment amplifications, using ET Dye Terminator Sequencing Chemistry (GE Healthcare Life Sciences). Non-incorporated dyes were removed using Sephadex G-50 fine (Amersham Biosciences, Sweden), and the sequencing reaction products were separated on a MegaBACE 500 automated capillary sequencer. Sequences were manually checked and edited using Codoncode aligner 3.03.

For the *fog* gene, we obtained sequences heterozygous for an indel, which precluded analysis for the region covered by the indel in these individuals. The sequence upstream and downstream of the indel could be inferred from the complementary strand. All sequences obtained in this study were deposited in GenBank (Accession Numbers: HM630611-HM631626).

### Sequence analysis

The sequences of the four mtDNA gene fragments (*mt:CoI*, *mt:ND4*, *mt:ATPase6* and *mt:ND5*) were concatenated prior to analysis. Sequences were aligned with MacClade 4.08 (Maddison & Maddison 2000). Standard diversity estimates were calculated with DnaSP version 5.10 (Librado & Rozas 2009).

We detected several heterozygous individuals for the nuclear genes. Their sequences were treated as follows. First, we inferred the phase of the haplotypes using the PHASE v2.1.1 program (Stephens *et al*. 2001; Stephens & Donnelly 2003) implemented in DnaSP. Analogue to previous treatments of inbred lines for diversity surveys, we randomly discarded one of the alleles in heterozygous individuals from inbred isofemale lines. In contrast, for the first generation offspring of freshly collected females, we kept both alleles. The mtDNA of three individuals (MAU22, MAU38 and RB12) showed evidence of heteroplasmy (maI and maII alleles). In all fragments, the most abundant haplotype based on the chromatogram peaks was maI for all three individuals; therefore, we score them as such. Two individuals (RED30 and KIB4) were heteroplasmic at a single position of their sequences, probably resulting from a new mutation occurring only in those individuals. In these two cases, we randomly discarded one of the alleles.

We assessed the phylogenetic relationship between the haplotypes present in each of the four data sets (i.e. the three nuclear fragments and the concatenated mtDNA fragment) using the maximum-likelihood method implemented in PAUP* 4.10b (Swofford 1998). The following mtDNA sequences available at GenBank were added to the mtDNA concatenated data set: DmelOregR/AF200828, DmelZim53/AF200829, Dsech/AF200832, DsimHW00/AF200835, DsimHW09/AF200836, DsimTT01/AF200834, DmauG52/AF200830, DsimAU023/AY518674, DsimC167/AF200839, DsimKY007/AY518670, DsimKY045/AY518671, DsimKY201/Y518673, DsimMD106/AF200842, DsimMD199/AF200852 and DmauBG1/AF200831. The reference sequences of *D. melanogaster* and *Drosophila sechellia* were added to the nuclear genes data sets.

Prior to analyses, we estimated the model of evolution that best fitted each data set using ModelTest v3.7 (Posada & Crandall 1998). For each data set, 100 bootstrap replicates were performed to assess nodes' support values. The final trees were displayed in Treeview (Page 1996). We used *D. melanogaster* as outgroup for these analyses.

### IM analysis

We used the software IM (Hey & Nielsen 2004), which considers a model of isolation with migration to estimate the number of migrants per generation between *D. mauritiana* and *D. simulans* (m), the time of split between the two species (t), their correspondent effective population sizes (Ne) and that of their common ancestor (NA). For this analysis, we assumed uninformative priors for the six model parameters under the HKY model for a data set consisting of the four sequenced loci, i.e. the mtDNA locus and the three nuclear loci. We ran the analysis multiple times under different heating schemes for the Metropolis coupled Markov chains until good mixing was achieved as determined by the parameters low autocorrelation over the course of the runs and by large values of the effective sample size estimate (above 10 000). The final analysis was repeated ten times for each data set to assess repeatability of the results using 100 Markov chains following a geometric heating scheme with 2 000 000 steps for the burn-in and 1 000 000 steps for the data collection. We considered a substitution rate of 1.54 × 10^−8^ per site/year/bp for the nuclear loci (Li 1997) and of 1.6 × 10^−8^ per site/year/bp for the mitochondrial DNA (Sharp & Li 1989). To account for the mutation rate uncertainty, we allowed the values to range between +/− one order of magnitude around the values mentioned earlier. We assumed ten generations per year.

### Timing of the split between D. mauritiana and D. simulans

We estimated the time to the most recent common ancestor (TMRCA) of all *D. mauritiana pcl* alleles, using only derived silent polymorphisms (11 out of a total of 154 silent sites), as a proxy of the minimum time of divergence between *D. mauritiana* and *D. simulans*. The TMRCA of those sequences was estimated using the software Genetree (Bahlo & Griffiths 2000) assuming a constant population size and ten generations per year. For the TMRCA estimation, we used (i) the estimated value of theta (θ = 3N_e_μ) calculated with Genetree for the *D. mauritiana* data and (ii) an estimated θ based on a published silent sites substitution rate of 1.54 × 10^−8^ per site/year/bp (Li 1997) and assuming a population size in *D. mauritiana* of one million individuals. For each value of θ, we ran Genetree (Bahlo & Griffiths 2000) three separate times with different random seeds and for one million coalescent simulations.

### Microsatellite analyses

Standard population genetics summary statistics (e.g. Heterozygosity, *F*_ST_) were calculated using msa v.4.1 (Dieringer & Schlötterer 2003). As the data set included inbred isofemale lines, we accounted for the random loss of allelic variation because of inbreeding by calculating the mean of 200 data sets of randomly discarded alleles. The allelic richness was estimated accounting for the unevenness in sample size between the two species by rarefaction analysis of the *D. mauritiana* samples to match the sample size in *D. simulans*.

A group-based analysis of population structure was performed with BAPS 5.2 (Corander & Martinen 2006) using the total number of groups analysed as the prior vector of the number of clusters present in the data and repeating the analysis to confirm repeatability of the results. In an initial step, the three samples collected during 2007 were analysed separately to determine the current population structure in Mauritius. As these samples clustered together with a posterior probability of 0.94, they were pooled as a single group for the remaining analyses. We calculated a neighbour-joining (NJ) tree in Phylip 3.6b (Felsenstein 1989) based on the distance – proportion of shared alleles (Bowcock *et al*. 1994) – between samples pooled by collection year. A total of 100 bootstrap replicas were performed to assess node support. The final tree was displayed in Treeview (Page 1996).

We determined a null distribution of the statistic *F*_ST_ to identify outlier loci (outside the 95 percentile of the distribution) that could reflect genomic regions permeable or refractory to introgression. For this purpose, we simulated 10 000 loci under the assumptions of neutrality and demographic equilibrium using the program *ms* (Hudson 2002). We assumed a θ estimate of four (i.e. the average of the θ estimates for *D. mauritiana* and *D. simulans* inferred from the observed gene diversity assuming the stepwise mutation model and after correcting the X-linked markers'θ estimate by 4/3 to account for the X chromosome's smaller Ne), a diploid Ne of one million individuals, ten generations per year and a divergence time between *D. mauritiana* and *D. simulans* of 250 000 years ago (McDermott & Kliman 2008) (the ms command used was: ./ms 400 10000 -t 4 -I 2 200 200 -ej 1.25 1 2). To accommodate the uncertainty in the divergence time between the species, we also performed this analysis for the extremes of the 95% confidence intervals (95%CI) of the divergence time estimated by McDermott & Kliman (2008), i.e. lower bound of the 95%CI ∼50 000 and upper bound of the 95%CI ∼510 000 years ago. The output of the ms program was converted to microsatellite data following the stepwise mutation model with the script ms2ms.pl (Pidugu & Schlotterer 2006).

## Results

### Nuclear loci show complete monophyletic clustering of Drosophila simulans and Drosophila mauritiana individuals

#### Nuclear genes

We sequenced DNA fragments of three nuclear genes: *pcl*, *fog* and *mav*. The phylogeny of the three species of the *D. simulans* complex differs among the three nuclear gene fragments (Fig. S1). This result is well described and illustrates the difficulties in solving the species relationships when lineages split recently (Caccone *et al*. 1988, 1996; Singh 1989; Hey & Kliman 1993; Kliman & Hey 1993; Hilton *et al*. 1994; Harr & Schlötterer 2004). Nevertheless, for all loci, the *D. simulans* and *D. mauritiana* sequences show monophyletic clustering (Fig. S1). In fact, the only single polymorphic site shared between *D. mauritiana* and *D. simulans* occurred in the *fog* locus. Pairwise nucleotide diversity for *mav* and *fog* is approximately one order of magnitude higher in *D. simulans. pcl* is completely monomorphic in *D. simulans*, while *D. mauritiana* has almost ten times higher levels of polymorphism than in the other loci (Table 1).

**Table 1:** Estimates of polymorphism and divergence for the three nuclear loci

	*S*	π	*F*	S*	Ks
*pcl* (595 bp)					
*Drosophila mauritiana*	24	0.0015	7	0	0.021
*Drosophila simulans*	0	0.0000			
*fog* (479 bp)					
*D. mauritiana*	15	0.0003	4	1	0.015
*D. simulans*	11	0.0088			
*mav* (582 bp)					
*D. mauritiana*	5	0.0005	6	0	0.025
*D. simulans*	4	0.0029			

Sample size for *D. mauritiana* = 165 and for *D. simulans* = 31. Sequence length, after discarding alignment gaps, is shown in brackets after the gene name. *S* is number of segregating sites; π is silent nucleotide diversity with Jukes-Cantor correction; *F* is the number of fixed differences between *D. mauritiana* and *D. simulans*; S* is the number of shared polymorphisms between *D. mauritiana* and *D. simulans*; Ks is the average number of silent substitutions per site between *D. mauritiana and D. simulans*.

*pcl* is located in a region of reduced recombination rate in *D. melanogaster* and *D. simulans*, but nonreduced in *D. mauritiana*. Because this is a derived state in *D. mauritiana*, the increased variability of *pcl* in *D. mauritiana* relative to *D. simulans* probably results from mutations that occurred after their split. Therefore, we considered the time to TMRCA of the derived mutations as a lower boundary of the species isolation time. We obtained two estimates of the TMRCA depending on the θ used. While the TMRCA estimated based on the published silent substitution rate of 1.54 × 10^−8^ for *D. melanogaster* (θ = 0.712) was 299 080 years, the one estimated from our data (θ = 1.935) resulted in a minimum divergence time of 210 400 years. The three replicates of each analysis yielded consistent results.

#### Microsatellites

**Fig. 1:**
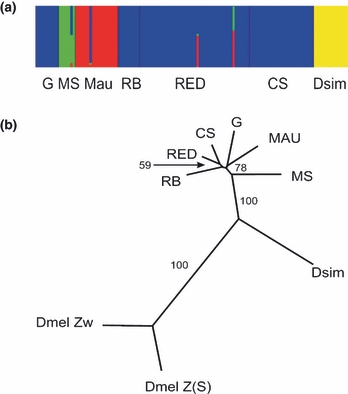
Multilocus microsatellite data show complete separation between *Drosophila simulans* and *Drosophila mauritiana* individuals. (a) Group-based Bayesian analysis of population structure. Collections are separated by black bars. Admixed individuals show more than one colour representing by the colour's proportion in the bar the proportion of ancestry from a particular cluster. (b) Neighbour-joining tree between the collections of *D. mauritiana, D. simulans* and *Drosophila melanogaster*. Only bootstrap support values above 75 are shown.

*Drosophila* microsatellites evolve slowly (Schug *et al*. 1997; Schlötterer *et al*. 1998; Bachtrog *et al*. 2000) but their mutation rates are still higher than those of single-copy genes, making them useful markers to study relationships among closely related species (Harr *et al*. 1998). Microsatellite polymorphism varied between a maximum of 16 alleles and a minimum of one with an average of 7.84 alleles per locus, and the average expected heterozygosity ranged from 0.301 to 0.396 (Table S3). The 4th chromosome marker was fixed for the same allele in both species, and overall, *D. mauritiana* presented a higher allelic richness than *D. simulans* (4.15 vs. 3.2 alleles per locus, respectively). The BAPS analysis yielded four clusters supported with a posterior probability of one, separating *D. mauritiana* from *D. simulans*. The collections MAU and MS formed separate clusters from the remaining *D. mauritiana* samples. Remarkably, the third *D. mauritiana* cluster included the 1979 (G) sample and the more recent samples collected since 2000 (RB, RED and CS) indicating the lack of temporal isolation between these collections. Four *D. mauritiana* individuals from the MS, MAU and RED collections were significantly admixed (Fig. 1a). No admixture between the two species was detected.

The pairwise *F*_ST_ comparison between collections and their correspondent *p-value* after Bonferroni correction are shown in Table S4. Within *D. mauritiana*, one-third of the pairwise comparisons are significant and in all cases these involve a comparison with MAU or MS reflecting the higher differentiation of the last two clusters compared to the remaining *D. mauritiana* samples as observed with the BAPS analysis. We cannot distinguish whether this differentiation results from particular natural history features of these samples or whether this is attributable to the kinship between the lines (e.g. MS lines share a higher proportion of alleles than the other populations). The fact that these are inbred lines excluded the possibility of using kinship tests, as it is not trivial to account for the random loss of alleles during the inbreeding process. As observed earlier, no significant temporal differentiation was observed between the G sample of 1979 and the most recent samples of 2007 despite of the approximately 280 generations separating them. All comparisons between species are significant and reflect high differentiation. In concordance with the lack of admixture between *D. mauritiana* and *D. simulans* found in the BAPS analysis, the NJ tree calculated based on the proportion of shared alleles separated both species with a bootstrap support of 100% (Fig. 1b). Within *D. mauritiana*, only the MS collection presented a bootstrap support higher than 75% separating it from the remaining sample sets. The lack of high bootstrap support values for the remaining branches within the *D. mauritiana* clade reflects the little differentiation between sample sets observed with the *F*_ST_ analysis.

### Paraphyletic clustering of D. simulans and D. mauritiana mtDNA sequences and identification of a new mtDNA haplotypic class in D. mauritiana

We obtained approximately 2 kb of mtDNA sequence from eight collections of *D. mauritiana* sampled over a period of at least 28 years (Table S1 and S5). We also included 19 *D. simulans* individuals from populations located at the centre of the species diversity (Table S1 and S5), where all haplotypic classes can be found (Solignac 2004). Ten additional haplotypes were found in sequences from GenBank and they were also included in the analyses.

**Fig. 2:**
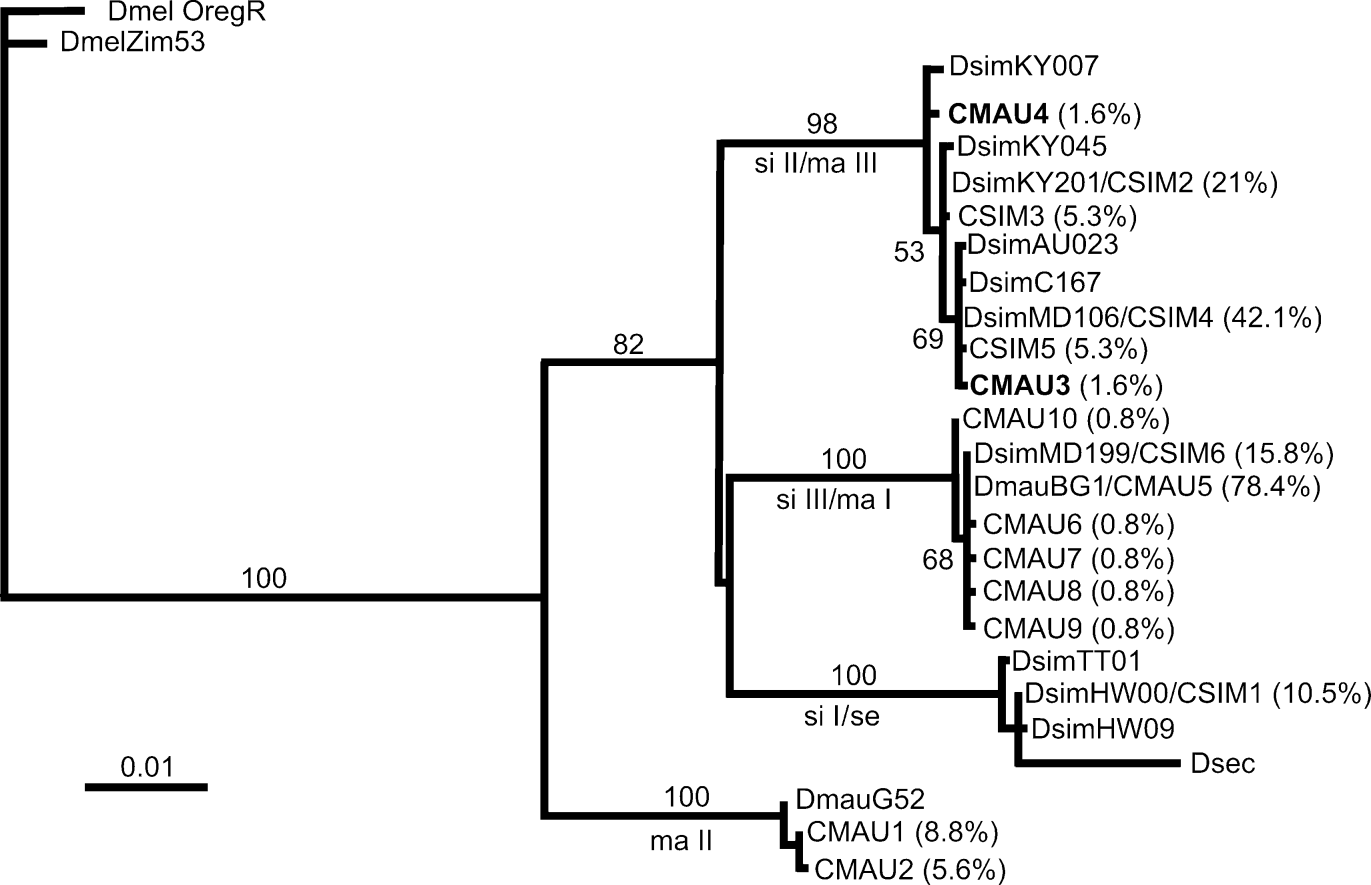
Maximum-likelihood phylogeny of mtDNA haplotypes of the *Drosophila melanogaster* subgroup, based on four mtDNA fragments. The new maIII *Drosophila mauritiana* haplotypes are shown in bold. Haplotypes obtained from GenBank (see Materials and Methods) carry a prefix indicating the species origin, whereas haplotypes identified in this study have the prefix CMAU or CSIM, depending on whether they were isolated from *D. mauritiana* or *Drosophila simulans* individuals, respectively. Numbers inside the brackets are the frequency of each haplotype in the correspondent species. Bootstrap values, above branches, are calculated based on 100 replicates and indicate the statistical support for the corresponding node. The haplotypic class of a given branch is indicated below the branches.

Figure 2 shows the phylogenetic relationships between the haplotypes found for the sequenced mtDNA region. The clear differentiation between *D. simulans* and *D. mauritiana* observed at nuclear loci is no longer apparent for the mtDNA. As previously reported, mtDNA sequences do not cluster according to their species identity (Solignac *et al*. 1986; Satta & Takahata 1990; Ballard 2000a), with individuals having mtDNA haplotypes that more closely related to those of the other species than to those of its own. Separation between the major haplotypic groups is supported by high bootstrap support values for the corresponding nodes. In total, ten different haplotypes were found in *D. mauritiana*. Most of these haplotypes (about 60%) fall into the siIII/maI haplogroup, which is the most abundant one in our data set (about 82%, Fig. 2). In the highly divergent maII haplogroup, which is endemic to Mauritius (Solignac 2004), we identified three different haplotypes. One of them, GenBank accession DmauG52, is absent from our collections. Almost 15% of the individuals in our collection harbour mtDNA of the maII type. The relative frequencies of maI and maII in our collections are similar to previous studies (Aubert & Solignac 1990).

Interestingly, we detected two new mtDNA haplotypes (highlighted in Fig. 2) that do not fall into any of these two haplotypic groups. The new variants clustered with *D. simulans* sequences of the siII haplogroup. These haplotypes are present in only four of the 125 *D. mauritiana* individuals (Mau17, Mau24, RB11 and RED25) and so far only *D. simulans* individuals were known to carry this mtDNA type. In this work, we refer to this new *D. mauritiana* haplotype group as maIII.

The frequency of the three different haplotypic groups is shown in Fig. S2. Two collections, MAU and MS, stand out from the rest by showing a much higher proportion of maII, (44 and 86% respectively). After the removal of MAU and MS, there is no difference between collections in the frequency of the three haplotype classes (*P* > 0.3, contingency table χ^2^ statistical test). While the observed differences in frequencies of the different haplotypes can result from a higher relatedness of MAU and MS (see Materials and Methods), it is also possible that these collections are truly differentiated from the others because of, for example, unrecognized temporal structure in *D. mauritiana*.

Given the known high divergence between haplotypic groups, it is not surprising to find considerable mtDNA diversity within species (Table 2). As previously described for the *D. simulans* clade, variability drops significantly within haplotypic classes. Interestingly, maIII harboured the highest amount of polymorphism, being almost two times more polymorphic than the corresponding *D. simulans* siII haplotypes. This is particularly interesting, as siII is the only haplotype for which no reduced variability has been detected in *Wolbachia*-free *D. simulans* populations (Ballard 2004). It is also worth noting that maI shows higher diversity than siIII. While sampling variance could be responsible for this difference, a large *D. simulans* population survey in regions where siIII is common also found very little polymorphism in this class (Dean *et al*. 2003).

**Table 2:** Estimates of variability for each haplotypic class

	*n*	*S*	*h*	Hd	π
*Drosophila mauritiana* (2088 bp)					
maI	103	6	6	0.10	0.00004
maII	18	1	2	0.50	0.00104
maIII	4	6	2	0.67	0.00699
Total	125	112	10	0.38	0.04793
*Drosophila simulans* (2088 bp)					
siI	2	0	1	0.00	0.00000
siII	14	5	4	0.63	0.00370
siIII	3	0	1	0.00	0.00000
Total	19	94	6	0.78	0.05934

Sequence length, after discarding alignment gaps, is shown in brackets after the species name. *n* is sample size; *S* is number of segregating sites; *h* is number of haplotypes; Hd is haplotype diversity; π is silent nucleotide diversity with Jukes-Cantor correction.

The high sequence similarity between maIII and siII may be the result of a hitherto unrecognized event of introgression because of the hybridization between *D. mauritiana* and *D. simulans*. Alternatively, their shared variation could result from ancestral polymorphism still segregating in the descendant lineages. These two alternative scenarios are discussed later.

### IM analyses- isolation with or without migration?

Given the high amount of shared polymorphism between *D. simulans* and *D. mauritiana* in the mtDNA data and almost entirely fixed differences at the nuclear loci, we were interested to know whether the data could be explained by a simple isolation model.

**Fig. 3:**
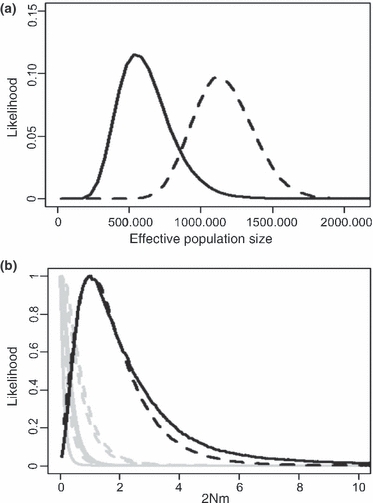
Marginal posterior distribution of the parameters estimated under the IM model. (a) Effective population size of *Drosophila mauritiana* (dashed line) and *Drosophila simulans* (solid line). (b) Effective number of migrants per generation into *D. mauritiana* (dashed lines) and into *D. simulans* (solid lines) for the mtDNA (black) and for the three nuclear loci (grey).

Using the IM software, we obtained consistent estimates across independent replicas for the N_e_ of *D. mauritiana* and *D. simulans*, and the number of migrants into each of the species. Estimates of the effective population size of the ancestral population (NA) and *t* were not convergent across replicates or the posterior distribution was flat and uninformative reflecting the lack of information content about these parameters in our data set. Our data shows that *D. mauritiana* has an N_e_ almost twice as large as that of *D. simulans*, 1.129 375 and 552 027, respectively (the reported values are means across 10 replicates of the analysis). The migration rate estimate revealed extensive migration at the mtDNA level but none at the nuclear DNA level (Table 3, Fig. 3, and Supporting Table S6).

**Table 3:** Parameter estimates obtained from IM

		2 Nm			
		
Species	Ne	mtDNA	mav	fog	pcl
*Drosophila mauritiana*	1 129 375 (633 052–1 645 952)	1.07 (0.014–3.74)	0.014 (0.01–0.322)	0.014 (0.01–0.52)	0.014 (0.01–0.462)
*Drosophila simulans*	552 027 (227 908-1 088 869)	1.08 (0.089–5.00)	0.007 (0.01–0.294)	0.026 (0.01–0.62)	0.054 (0.01–0.68)

Ne is the effective population and 2 Nm is the effective migration rate per generation calculated for each locus. In parenthesis are the minimum and maximum boundaries of the 90% highest posterior density for the estimated parameter. Migration rate estimates reflect migration rates into the corresponding species. The parameter estimates provided in this table are the estimates' average across 10 replicate analyses, and the HPD values are the most extreme ones found across these replicas.

### The introgression of siII into D. mauritiana occurred in the last 125 000 years

Because we found evidence of gene flow for the mtDNA between the two species, we estimated the time of the putative introgression based on the number of observed silent site changes between the closest *D. simulans s*iII- *D. mauritiana* maIII sequence pair. Figure 2 illustrates that DsimKY007 is the closest sequence to MAU4, whereas several haplotypes are equally distant to MAU3. To increase the number of informative sites and decrease the error in our estimates, we sequenced two additional mtDNA fragments from the four *D. mauritiana* individuals bearing the new haplotypes (see Materials and Methods). In total, the six concatenated mitochondrial fragments contained 737 silent sites. We found three silent site substitutions between *D. mauritiana* MAU4 and its closest *D. simulans* relative DsimKY007. Assuming a silent substitution rate of 1.6 × 10^−8^ (Sharp & Li 1989) per site per year, the mean divergence time between these two haplotypes is 127 205 years and the maximum time of divergence compatible with observing no more than three mutations is 325 000 years (*P* < 0.05, Poisson distribution). There were no silent differences between MAU3 and several *D. simulans* sequences (i.e. DsimMD106). This implies a split no longer than 125 000 years ago (*P* < 0.05, Poisson distribution).

### MtDNA introgression- one, two, three?

If we accept that MAU4 is not an ancestral allele segregating in *D. mauritiana*, then its sequence could be derived from MAU3 after this haplotype introgressed from *D. simulans* into *D. mauritiana*. However, as the distance between MAU3 and MAU4 is larger (five synonymous substitutions) than that between MAU4 and DsimKY007 (three synonymous substitutions, Fig. 2) the divergence between the two maIII haplotypes dates well longer than 325 000 years ago. Therefore, the occurrence of MAU3 and MAU4 in *D. mauritiana* probably resulted from independent introgression events, either simultaneously or at different time points. These results imply that in addition to the described introgression involving the siIII/maI haplotypes, one and possibly two additional events of mtDNA introgression must have occurred in the recent history of these species.

### Does Wolbachia facilitate cytoplasmic introgression?

The movement of mtDNA across species boundaries might be driven by *Wolbachia-*induced CI or beneficial effects of *Wolbachia* on its host (Ballard 2000b; Hurst & Jiggins 2005; Bachtrog *et al*. 2006). We sought to determine whether maIII individuals are infected with the same *Wolbachia* strain that occurs in those *D. simulans* individuals with the siII mtDNA type. According to predictions based on a study by Ballard (2004), flies with maIII-MAU4 haplotype should have no infection because DsimKY007 belongs to a clade of siII haplotypes not infected with *Wolbachia* while maIII-MAU3 individuals could be uninfected or infected with wRi or wAu *Wolbachia*. However, our screen for *Wolbachia* showed that none of the four individuals with maIII mtDNA was infected (Table S7).

### Analysis of individual microsatellites reveals an excess of significantly high and low differentiated loci

Unless introgression following hybridization involves preferentially the mtDNA, we would expect to see a signal of introgression also in the nuclear data. While the combination of several microsatellites is expected to give a robust signal of the species divergence, individual loci could potentially reveal localized events of gene flow between species.

**Fig. 4:**
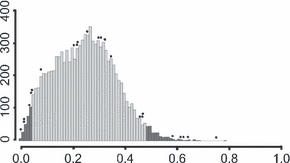
*F*_ST_ null distribution for 10 000 loci. The 95 percentile of the distribution is in light gray and the 5 percentile extreme values are highlighted in dark gray. The black dots represent the *F*_ST_ values for each of the microsatellites. The *F*_ST_ value for the marker on the 4th chromosome is not shown because the same allele is fixed in both species. Counts refer to the number of times that each *F*_ST_ value was observed in the simulations.

Our analysis of the microsatellite data identified an excess of markers that presented a lower or a larger than expected *F*_ST_ when compared to that statistic's null distribution (Fig. 4). As the 4th chromosome microsatellite was fixed for the same allele in both species, it was not considered for this analysis. Considering that 5% of the markers could randomly show an extreme *F*_ST_ value, we expected no more than one locus showing such an extreme value. Contrary to our expectation, we found that five microsatellites showed an *F*_ST_ smaller than the lower 95% cut-off of our estimated *F*_ST_ null distribution (two loci on the 2nd chromosome and three on the 3rd chromosome). We also found six microsatellites with a larger than expected *F*_ST_ value (three loci on the X chromosome, two on the 2nd chromosome and one on the 3rd Chromosome). In neither case are these loci characterized by a reduced or a large heterozygosity. In both cases, the mean locus heterozygosity is 0.28 with a minimum of 0.08 for both sets of loci and a maximum of 0.48 for the low *F*_ST_ loci and 0.41 for the high *F*_ST_ loci. As the maximum value the *F*_ST_ can reach can be biased when extremely polymorphic markers like microsatellites are used, we repeated our analysis with the statistic *G*_ST_' which is standardized to account for large heterozygosity values (Hedrick 2005). We confirmed our results with the *F*_ST_ analysis but instead of finding five loci with extremely low *G*_ST_' values, we found ten such outlier markers. As the maximum *G*_ST_' observed in our data set and in the simulations was one, there were no outliers with unexpectedly large *G*_ST_' values. Repeating this analysis for the extremes of the 95% confidence interval of the divergence time between these species (McDermott & Kliman 2008) reduced the number of loci with a lower than expected *F*_ST_ from five to three for the lower 95%CI of tau (∼50 000 years), while for the upper 95%CI of tau (∼510 000 years) the same six loci with a higher than expected *F*_ST_ remained as outlier. The equivalent analysis for the *G*_ST_' reduced by one locus the number of loci with a lower than expected *G*_ST_'.

## Discussion

### mtDNA introgression

Gene flow between *D. mauritiana* and *Drosophila simulans* is currently supported mostly by the existence of a shared mtDNA haplogroup, maI-siIII (Solignac & Monnerot 1986; Ballard 2000b). Analyses of gene flow are extremely difficult, and its inference based on a single type of analysis is likely to be unreliable. Hence, we have used multiple pieces of evidence to support our conclusion that at least one, but possibly two additional independent mtDNA introgression events occurred. Based on *pcl*, we were able to infer that *D. simulans* and *D. mauritiana* must have diverged from a common ancestral pool at least 210 000 years ago. It is possible that under certain circumstances the time of coalescence of the *D. mauritiana pcl* alleles could be deeper than the time of species split. However, McDermott & Kliman (2008) have recently re-estimated the split between *D. simulans* and *D. mauritiana* to have occurred around 250 000 years ago, well within the range of values obtained by us. Given these estimates of the speciation time, the occurrence of the MAU3 haplotype in *D. mauritiana* is more likely to be explained by recent gene flow between the two species than by segregating ancestral polymorphism.

We cannot rule out that MAU4 is an old ancestral allele segregating at low frequency in *D. mauritiana*. However, given the overlap between the estimates and the possibility that we have not sampled all *D. simulans* siII haplotypes (Ky007 belongs to a clade within siII particularly variable, Ballard 2004), it seems reasonable to suggest that the presence of MAU4 in *D. mauritiana* is the consequence of *another* introgression event. As mentioned before, it is unlikely that both MAU3 and MAU4 result from the same introgression event, because the accumulation of differences between them implies a divergence larger than the isolation time between the species.

Another two alternative scenarios could explain the observed patterns. First, contamination with *D. simulans* could have occurred during laboratory maintenance of the stocks. However, one of the individuals harbouring the maIII haplotype (RB11) was the F1 of a wild-collected female, rendering this hypothesis very unlikely. Furthermore, we detected no evidence for introgression for these lines using microsatellites and nuclear DNA sequence data. Recurrent mutation may also account for the observation, but given the recent species divergence, we do not consider this a plausible explanation.

In the largest published collection of *D. mauritiana* (345 individuals Aubert & Solignac 1990) only maI and maII haplotypes were found. The chance of not sampling maIII, given the frequency observed in our collections (4/125 individuals), is extremely small (*P* < 0.001, binomial probability mass function). The fact that despite intensive sampling efforts these two new haplotypes have not been detected raises the possibility that the admixture events may be of very recent origin. Nevertheless, we also note that seasonal fluctuations or population structure may also contribute to the absence of mauIII in previous collections.

### Direction of introgression

Our IM analysis supports the introgression of mtDNA from *D. mauritiana* into *D. simulans*. However, it has previously been proposed that introgression of mtDNA occurred from *D. simulans*, possibly from Madagascar or Réunion, to *D. mauritiana* (Solignac & Monnerot 1986; Aubert & Solignac 1990). This assumption is in part owing to the species distribution (*D. mauritiana* is an island endemic while *D. simulans* is cosmopolitan, although absent from Mauritius). In addition, experimental interspecific crosses are highly asymmetrical, e.g. insemination occurs more than 18 times more frequently when *D. simulans* is the female (Robertson 1983). Furthermore, introgression experiments found that the mtDNA of a single *D. simulans* female introduced into a vial of *D. mauritiana* at a frequency of 0.03 quickly became more abundant than the original *D. mauritiana* mtDNA and reached fixation in almost all populations tested. In contrast, the reciprocal experiment never led to the fixation of the *D. mauritiana* mtDNA (Aubert & Solignac 1990).

*Wolbachia* was also suggested to favour this direction of introgression because the *D. mauritiana* endemic maII is not infected (Ballard 2000b). MaI *D. mauritiana* individuals are infected with the same strain of *Wolbachia* (wMa) as siIII *D. simulans* flies (James & Ballard 2000). A *Wolbachia*-mediated sweep, because of either CI (although currently wMa does not seem to be able to induce strong CI, it is possible that at the time of introgression this was the case) or a fitness benefit bestowed to the host, could have facilitated mtDNA introgression from *D. simulans* to *D. mauritiana*, because hybrid flies with the ‘migrant cytoplasm’ would have an advantage over pure-species individuals. While this might be a reasonable hypothesis in the maI-siIII case, it is very unlikely that the two new introgression events detected in this study were driven by *Wolbachia*.

The alternative scenario, supported by our data, has also been considered before. Ballard (2000b) suggested that paternal leakage of mtDNA following a cross of a *D. mauritiana* male with *D. simulans* females could also explain the maI-siIII distribution. Field data on heteroplasmy (Dean *et al*. 2003) and experimental paternal leakage (Kondo *et al*. 1990) show that this might be a plausible explanation. In addition, as mentioned by Ballard (2000b), maI is the most abundant haplotype in *D. mauritiana* (88% in Solignac *et al*. 1983 and 82% in our data set, Fig. 2), but siIII is rare in *D. simulans* (only slightly higher than 33% in Madagascar and Reunion). Combined with the complete lack of variation in the siIII haplotype group (but not in maI), this observation indicates that maI could have originated in *D. mauritiana* rather than in *D. simulans*. With these alternative hypotheses in mind, we think that the possibility that *D. simulans* flies from Madagascar and/or Reunion might have acquired siIII mtDNA following introgressive hybridization with *D. mauritiana* migrants is realistic. However, the maIII-siII introgressions are very unlikely to have occurred from *D. mauritiana* to *D. simulans* because siII has a cosmopolitan distribution and harbours significant variation in some populations (Ballard 2004).

It is still unclear whether conclusions based on data obtained experimentally bear significance in a natural context. *D. mauritiana* and *D. simulans* exhibit several differences in their mating behaviour (Robertson 1983), e.g. *D. mauritiana* females will accept only *D. simulans* males if there are no conspecific males accessible. Attempts to map loci involved in these premating reproductive isolating mechanism have found a minimum of three to eight QTLs with moderate to large effects in the X, 2nd and 3rd chromosomes (Moehring & Mackay 2004). However, mating behaviour in the laboratory might be very different from that in the natural environment and somehow conditions might be relaxed. For example, in the laboratory, *D. melanogaster* will mate with *D. mauritiana* in only 3% of the cases and only when *D. melanogaster* is the female. Despite this, a *D. melanogaster* female fertilized by *D. mauritiana* was detected in a collection expedition to Mauritius (Lachaise *et al*. 1988).

### Mitochondrial vs. nuclear introgression

Mitochondrial introgression has been inferred in a number of species and seems to occur more frequently than introgression of the nuclear. Despite intense speculation, the reasons for this difference were not really understood (Coyne & Orr 2004; Bachtrog *et al*. 2006) until recent work by Currat *et al*. (2008). They show that introgression is stronger for genes that experience smaller intraspecific gene flow because they would not ‘dilute’ introgressed genes from the invading population. While our data show a convincing signal of recurrent mtDNA introgression, the conclusions to be drawn from the nuclear data are more speculative. Despite the lack of admixture between individuals of the two species based on 25 microsatellite loci, a detailed locus by locus analysis revealed five loci with unexpectedly low differentiation values. As these markers showed no evidence for reduced levels of polymorphism, these loci might reflect genomic regions permeable to gene flow between the species, rather than regions subject to selection or reduced recombination rate. We also detected several loci with an unexpectedly high *F*_ST_ value. Fifty per cent of these loci were located on the X chromosome, but the interpretation of these extreme divergence values is not clear and may simply reflect an inadequate population model used in our simulations.

## Conclusions

In this work, we have identified two new haplotypes in *D. mauritiana* that cluster with the siII haplotypic group of *D. simulans*. We have shown that the presence of these two haplotypes is more likely to result from introgression of mtDNA from *D. simulans* to *Drosophila mauritiana* than from extant ancestral polymorphism. This finding raises the number of detected mtDNA introgression events in the *D. simulans* clade to three, which means that at least three independent hybridization events must have occurred in the history of these species. We also provided evidence supporting the symmetrical mitochondrial gene flow between the species, which contradicts empirical data showing strong asymmetry of hybridization in experimental crosses. This might be suggestive of the behaviour of the flies in the laboratory being significantly different from their behaviour in nature.

If hybridization is such a frequent event, why are these species reciprocally monophyletic at the nuclear level? How many loci involved in reproductive isolation are necessary to keep two species apart? What genes are involved? Which regions can overcome the species barrier without any significant impact on the fitness of hybrids? What is the relative importance of selection and drift during this process? Only now, we are starting to have the adequate data to answer these questions. Some genes involved in hybrid male sterility or inviability have finally been identified (Wittbrodt *et al*. 1989; Ting *et al*. 1998; Barbash *et al*. 2003; Presgraves *et al*. 2003; Brideau *et al*. 2006; Masly *et al*. 2006; Tang & Presgraves 2009). One of the major findings is that natural selection plays a major role in shaping the evolution of these loci (Wu & Ting 2004; Tang & Presgraves 2009).

Massively parallel sequencing data have the potential to finally provide the amount of data necessary to answer these questions.

## Supporting information

**Fig. S1** Phylogenetic relationship between *fog* (A), *pcl* (B) and *mav* (C) haplotypes of the *D. melanogaster* subgroup.

**Fig. S2** Frequency distribution of the different mitochondrial groups per collection.

**Table S1** Collection details and samples sizes used for each analysis

**Table S2** List of primers used for amplification and sequencing for mitochondrial and nuclear genes, and microsatellites

**Table S3** Expected heterozygosity based on random discarded data sets

**Table S4** *F*_ST_ between collections

**Table S5** Sample identification labels and haplotypes

**Table S6** Parameter estimates of the IM model

**Table S7** Percentage of *Wolbachia* infected and uninfected individuals for a given fly collection
