# Supplementary material for: Multiple hybridization events between Drosophila simulans and Drosophila mauritiana are supported by mtDNA introgression
Source: Mol Ecol. 2010 Nov;19(21):4695–707. doi: 10.1111/j.1365-294X.2010.04838.x (PMC3035818; doi:10.1111/j.1365-294X.2010.04838.x)

# Supporting information

Table S1. Collection details and samples sizes used for each analysis.

Table S2. List of primers used for amplification and sequencing for mitochondrial and nuclear genes, and microsatellites.

Table S3. Expected heterozygosity based on random discarded datasets.

**Table S4.** Fst between collections.

**Table S5.** Sample identification labels and haplotypes.

Table S6. Parameter estimates of the IM model.

**Table S7.** Percentage of *Wolbachia* infected and uninfected individuals for a given fly collection.

**Figure S1.** Phylogenetic relationship between *fog* (A), *pcl* (B) and *mav* (C) haplotypes of the *D. melanogaster* subgroup.

Figure S2. Frequency distribution of the different mitochondrial groups per collection.

# Supplemental Material for Online Publication only

## Supplementary Tables

Table S1. Collection details and samples sizes used for each analysis.

| Label | Collection Place | Collection Year | Generation | 4mtDNA | 3nDNA | n microsatellites | n Wolbachia |
| --- | --- | --- | --- | --- | --- | --- | --- |
| *D. mauritiana* | | |  |  |  |  |  |
| G | Mauritius | 1979 | inbred | 12 | 12 | 10 | 0 |
| MS | Mauritius | 1987 | inbred | 7 | 7 | 7 | 0 |
| RB | Mauritius, Pointe aux Cannoniers | 2001 | F1 | 12 | 12 | 9 | 8 |
| RED | Mauritius, Reduit | 2006 | inbred | 48 | 48 | 46 | 8 |
| TAM | Mauritius, Tamarin | 2007 | F1 | 18 | 18 | 18 | 8 |
| LAB | Mauritius, Labordonnais | 2007 | F1 | 8 | 8 | 7 | 8 |
| CapM | Mauritius, Cap Malheureux | 2007 | F1 | 2 | 2 | 2 | 2 |
| Mau | Mauritius | unknown | inbred | 18 | 18 | 18 | 8 |
| *D. simulans* | | |  |  |  |  |  |
| Kam | Uganda, Kampala | 2000 | inbred | 2 | 2 |  |  |
| Kib | Uganda, Kibale forest | 2001 | F1 | 3 | 3 |  |  |
| Tun | Tunisia, Djerba | 2001 | F1 | 3 | 3 |  |  |
| ZOM | Malawi, Zomba | 2001 | F1 | 3 | 3 |  |  |
| SiTa | Tanzania, Arusha | 2006 | F1 | 3 | 3 |  | 2 |
| M | Madagascar | unknown | inbred | 3 | 3 |  |  |
| MS | Mauritius | 1987 | inbred | 2 | 2 |  |  |
| Np | Italy, Naples | 2000 | F1 |  |  | 1 |  |
| Chi | China, Jiamusi | 2002 | F1 |  |  | 2 |  |
| Evo | Portugal, Évora | 2004 | F1 |  |  | 3 |  |
| Cbr | Brazil, Campinas, São Paulo | 2005 | F1 |  |  | 2 |  |
| *D. melanogaster* | | |  |  |  |  |  |
| Z(S) | Zimbabwe, Sengwa | 1990 | inbred |  |  | 11 |  |
| Zw | Zimbabwe, Victoria Falls | 2001 | inbred |  |  | 21 |  |

n: number of individuals, 4mtDNA: mitochondrial DNA, 3nDNA: nuclear DNA.

Table S2. List of primers used for amplification and sequencing for mitochondrial and nuclear genes, and microsatellites.

| Target Gene | Target species | Primers | Annealing Temperature (°C) | Amplification Product size (bp) |
| --- | --- | --- | --- | --- |
| *mt:CoI* | *D. simulans* species complex | FW: 5’-GTAATTGTAACTGCACATGCTT-3’  RV: 5’-ATTCCTAAAGAACCAAAAGTTTC-3’ | 55 | 653 |
| *mt:ND4* | *D. simulans* species complex | FW: 5’-CAAGACGTTCATAAGATACATTAGC-3’  RV: 5’-GAGGTTATCAACCAGAACGTTT-3’ | 62 | 588 |
| *mt:Cyt-b* | *D. simulans* species complex | FW: 5’-CTTTAGTAGATTTACCAGCTCC-3’  RV: 5’-AAATATCATTCTGGTTGAATATG-3’ | 50 | 772 |
| *mt:ND2* | *D. simulans* species complex | FW: 5’-CTCCTTTCCATTTTTGATTTC-3’  RV: 5’-TGCAATTTTGAAGGAGTAATTT-3’ | 58 for D. sim  48 for D. mau | 794 |
| *mt:ND5* | *D. simulans* species complex | FW: 5’-CCTATTAAACGAATATCTTGAGA-3’  RV: 5’-TAGGACTTGTTTCTTATTGTTTAGT-3’ | 50 | 637 |
| *mt:ATPase6* | *D. simulans* species complex | FW: 5’-TCAGCTATTTTTAATTTATCACTT-3’  RV: 5’-GATTGAATTATAGCTACAGCTGATT-3’ | 50 | 593 |
| *pcl* | *D. simulans* species complex | FW: 5’-TGTTCTGAAACGTCGGGGT-3’  RV: 5’-GTATCGRAGGGCCGAGACA-3’ | 60 | 718 |
| *fog* | *D. simulans* species complex | FW: 5’-CCAGGCGATTTTCACGTTC-3’  RV: 5’-TGATGCCTTCTCCACATCG-3’ | 60 | 584 |
| *mav* | *D. simulans* species complex | FW: 5’-AACGGTGATGCACGTATTG-3’  RV: 5’-TACTACGTGGCRAACAGGA-3’ | 60 | 669 |
| *16S rRNA* | *Wolbachia* | FW: 5’- ACGGTACTCACAGAAGAAGT-3’  RV: 5’- CGTTAGCTGTAATACAGAAAGT-3’ | 52 | 383 |
| Xr1m8 | *D. melanogaster* | FW 5’-acgcaaaaacagatcgcatt-3’  RV 5’-aacgtacagcagccacagg-3’ | 60 | 275 |
| X2m2 | *D. melanogaster* | FW 5’-ttgaagattcgttattgctggt-3’  RV 5’-ctcggtggctattttgacg-3’ | 59 | 265 |
| Xr2m7 | *D. melanogaster* | FW 5’-cccgaaacaacaagaacagc-3’  RV 5’-ttgcagtgacatgaggtgttg-3’ | 60 | 145 |
| Xr3m5 | *D. melanogaster* | FW 5’-cgtcacacgtcaggacttt-3’  RV 5’-cgtgtgctagtggcttgtct-3’ | 58 | 225 |
| Xr3m8 | *D. melanogaster* | FW 5’-caaacagacacacccactcac-3’  RV 5’- accgccctcgagtcatt-3’ | 60 | 314 |
| Xr4m5 | *D. melanogaster* | FW 5’-atcgcccagctcagatca-3’  RV 5’-ggttcactgcgattcggtta-3’ | 61 | 134 |
| Xr4m10 | *D. melanogaster* | FW 5’-gagggaaattcaacgaagaac-3’  RV 5’-gggccaataggaaatatgc-3’ | 58 | 342 |
| Xr5m5 | *D. melanogaster* | FW 5’-gacttcgcgttcgaccag-3’  RV 5’-gcgcattcaagtcaaagtca-3’ | 60 | 163 |
| 2r1m4 | *D. melanogaster* | FW 5’-tgcgaatggaagtcgagtaa-3’  RV 5’-gtgcatgggtccacttgag-3’ | 60 | 255 |
| 2r1m6 | *D. melanogaster* | FW 5’-gatgcccaaccaagcaaat-3’  RV 5’-gtcgaaatgcccgaagaaat-3’ | 61 | 191 |
| 2r2m6 | *D. melanogaster* | FW 5’-ctcacggacgcagatacaga-3’  RV 5’-tgcagaggcagtagcaacat-3’ | 60 | 283 |
| 2r2m8 | *D. melanogaster* | FW 5’-agaataccagagcgcacaat-3’  RV 5’-tttctggccctcttctagg-3’ | 57 | 165 |
| 2r3m4 | *D. melanogaster* | FW 5’-cagcatttcgcttgatttga-3’  RV 5’-gctccatttagcacaaccaat-3’ | 59 | 252 |
| 2r4m1 | *D. melanogaster* | FW 5’-tttgcatttacaagaagtggtct-3’  RV 5’-gccacaaccgaaatacatca-3’ | 58 | 132 |
| 2r4m3 | *D. melanogaster* | FW 5’-tggaacccgatggataaact-3’  RV 5’-aactctttaaccgtgcttttgtt-3’ | 59 | 220 |
| 2r5m1 | *D. melanogaster* | FW 5’-aataatacaacaggcggagctt-3’  RV 5’-agccaatgcgaaaccaact-3’ | 59 | 101 |
| 3r1m5 | *D. melanogaster* | FW 5’-caatctcagacaacgatacacg-3’  RV 5’-gaggctcaaaggaagacagc-3’ | 59 | 225 |
| 3r2m5 | *D. melanogaster* | FW 5’-ccacattgctgctcttgttg-3’  RV 5’-tgaatctaccagccacatacca-3’ | 60 | 375 |
| 3r2m8 | *D. melanogaster* | FW 5’-caaagcgcctgatctatggt-3’  RV 5’-ctgctggatttggctgtgt-3’ | 60 | 191 |
| 3r3m1 | *D. melanogaster* | FW 5’-cagagttcgtcatcgtcctgt-3’  RV 5’-cagagttcgtcatcgtcctgt-3’ | 60 | 311 |
| 3r4m2 | *D. melanogaster* | FW 5’-cacagacaaacaggatcaaacag-3’  RV 5’-atcgcgaaatgccacaga-3’ | 60 | 105 |
| 3r4m6 | *D. melanogaster* | FW 5’-gtttgccttactagaagcttgatg-3’  RV 5’-ccagcactacaagggatctattc-3’ | 59 | 194 |
| 3r5m7 | *D. melanogaster* | FW 5’-cccagcaggcaggagac -3’  RV 5’-cattagtatcgcgctgcattt-3’ | 60 | 371 |
| 4r2m3 | *D. melanogaster* | FW 5’-tttgaggaaattcagtgctttg-3’  RV 5’-tgattctcggcctgaataatatg-3’ | 60 | 375 |

Table S3. Expected heterozygosity based on random discarded datasets.

| Locality | H expected |
| --- | --- |
| G | 0.378 |
| MS | 0.301 |
| RB | 0.365 |
| RED | 0.396 |
| CS | 0.385 |
| Mau | 0.381 |

**Table S4.** Fst between collections. Below matrix pairwise Fst values. Above diagonal p-values after Bonferroni correction. In bold all significant comparisons.

|  | G | MS | Mau | RB | RED | CS | Dsim | DmelZ(S) | DmelZw |
| --- | --- | --- | --- | --- | --- | --- | --- | --- | --- |
| G |  | n.s. | n.s. | n.s. | n.s. | n.s. | 0.0036 | 0.0036 | 0.0036 |
| MS | 0.13913 |  | 0.0144 | n.s. | 0.0036 | 0.0036 | 0.0036 | 0.0036 | 0.0036 |
| MAU | 0.06195 | 0.178706 |  | n.s. | 0.0036 | 0.0036 | 0.0036 | 0.0036 | 0.0036 |
| RB | 0.042769 | 0.149608 | 0.084428 |  | n.s. | n.s. | 0.0036 | 0.0036 | 0.0036 |
| RED | 0.013368 | 0.11913 | 0.051891 | 0.018918 |  | n.s. | 0.0036 | 0.0036 | 0.0036 |
| CS | 0.023991 | 0.128561 | 0.062777 | -0.00712 | 0.003457 |  | 0.0036 | 0.0036 | 0.0036 |
| Dsim | 0.360143 | 0.369714 | 0.356836 | 0.340766 | 0.356113 | 0.345893 |  | 0.0036 | 0.0036 |
| DmelZ(S) | 0.472883 | 0.501284 | 0.495211 | 0.463568 | 0.510801 | 0.500927 | 0.422336 |  | n.s. |
| DmelZw | 0.429302 | 0.452003 | 0.452893 | 0.42021 | 0.473715 | 0.459203 | 0.389783 | 0.016914 |  |

**Table S5. Sample Identification Labels and Haplotypes.**

| Sample_ID | MSA-matrix | Haplotype |
| --- | --- | --- |
| g122 | G | I |
| g130 | G | I |
| g18 | G | I |
| g197 | G | I |
| g24 | G | I |
| g29 | G | I |
| g38 | G | I |
| g71 | G | I |
| g74 | G | I |
| g76 | G | I |
| ms11 | MS | II |
| ms11-8 | MS | II |
| ms17 | MS | II |
| ms17-4 | MS | II |
| ms34 | MS | II |
| ms61 | MS | I |
| ms9 | MS | II |
| mau1 | Mau | I |
| mau14 | Mau | I |
| mau16 | Mau | II |
| mau17 | Mau | III |
| mau2 | Mau | I |
| mau21 | Mau | II |
| mau22 | Mau | I |
| mau23 | Mau | II |
| mau24 | Mau | III |
| mau26 | Mau | II |
| mau3 | Mau | I |
| mau31 | Mau | II |
| mau35 | Mau | II |
| mau36 | Mau | I |
| mau37 | Mau | I |
| mau39 | Mau | II |
| mau4 | Mau | I |
| mau41 | Mau | II |
| rb10 | RB | I |
| rb11 | RB | III |
| rb12 | RB | I |
| RB13 | RB | I |
| RB14 | RB | I |
| RB16 | RB | I |
| rb17 | RB | I |
| RB3 | RB | I |
| rb7 | RB | I |
| Red10 | RED | I |
| Red11 | RED | I |
| Red12 | RED | I |
| Red13 | RED | I |
| Red14 | RED | I |
| Red16 | RED | I |
| Red18 | RED | I |
| Red19 | RED | I |
| Red20 | RED | I |
| Red21 | RED | I |
| Red22 | RED | I |
| Red23 | RED | I |
| Red24 | RED | I |
| Red25 | RED | III |
| Red26 | RED | I |
| Red27 | RED | I |
| Red28 | RED | I |
| Red29 | RED | I |
| Red3 | RED | I |
| Red30 | RED | I |
| Red31 | RED | I |
| Red32 | RED | I |
| Red33 | RED | I |
| Red34 | RED | I |
| Red35 | RED | I |
| Red36 | RED | I |
| Red37 | RED | I |
| Red38 | RED | I |
| Red4 | RED | II |
| Red41 | RED | I |
| Red42 | RED | I |
| Red43 | RED | I |
| Red44 | RED | II |
| Red46 | RED | I |
| Red48 | RED | I |
| Red49 | RED | I |
| Red5 | RED | II |
| Red50 | RED | I |
| Red52 | RED | I |
| Red53 | RED | I |
| Red56 | RED | I |
| Red59 | RED | I |
| Red6 | RED | I |
| Red60 | RED | I |
| Red7 | RED | I |
| Red8 | RED | II |
| CapH39 | CapM | I |
| CapH40 | CapM | I |
| Lab1 | LAB | I |
| Lab2 | LAB | I |
| Lab4 | LAB | I |
| Lab5 | LAB | I |
| Lab6 | LAB | I |
| Lab7 | LAB | I |
| Lab8 | LAB | I |
| Tam1 | TAM | I |
| Tam11 | TAM | I |
| Tam12 | TAM | I |
| Tam13 | TAM | I |
| Tam14 | TAM | I |
| Tam15 | TAM | I |
| Tam16 | TAM | I |
| Tam17 | TAM | I |
| Tam18 | TAM | I |
| Tam19 | TAM | I |
| Tam20 | TAM | I |
| Tam21 | TAM | I |
| Tam22 | TAM | I |
| Tam3 | TAM | I |
| Tam5 | TAM | I |
| Tam6 | TAM | I |
| Tam7 | TAM | I |
| Tam8 | TAM | I |
| cbr3 | Cbr | na |
| cbr4 | Cbr | na |
| chi55 | Chi | na |
| chi57 | Chi | na |
| evo12 | Evo | na |
| evo13 | Evo | na |
| evo16 | Evo | na |
| evo27 | Evo | na |
| evo33 | Evo | na |
| evo34 | Evo | na |
| evo35 | Evo | na |
| evo39 | Evo | na |
| evo4 | Evo | na |
| evo44 | Evo | na |
| np4 | Np | na |
| zs22 | Z(S) | na |
| zs24 | Z(S) | na |
| zs28 | Z(S) | na |
| zs30 | Z(S) | na |
| zs35 | Z(S) | na |
| zs37 | Z(S) | na |
| zs40 | Z(S) | na |
| zs48 | Z(S) | na |
| zs49 | Z(S) | na |
| zs53 | Z(S) | na |
| zs56 | Z(S) | na |
| zw104 | Zw | na |
| zw106 | Zw | na |
| zw109 | Zw | na |
| zw114 | Zw | na |
| zw122 | Zw | na |
| zw123 | Zw | na |
| zw130 | Zw | na |
| zw136 | Zw | na |
| zw137 | Zw | na |
| zw140 | Zw | na |
| zw141 | Zw | na |
| zw142 | Zw | na |
| zw148 | Zw | na |
| zw149 | Zw | na |
| zw155 | Zw | na |
| zw164 | Zw | na |
| zw165 | Zw | na |
| zw168 | Zw | na |
| zw177 | Zw | na |
| zw184 | Zw | na |
| zw185 | Zw | na |

**Samples are ordered as in the MSA matrix. na: not available.**

Table S6. Parameter estimates of the IM model.

|  |  | Ne | | 2Nm into *D.mauritiana* | | | | 2Nm into *D.simulans* | | | |
| --- | --- | --- | --- | --- | --- | --- | --- | --- | --- | --- | --- |
| replica |  | *D.mauritiana* | *D.simulans* | mtDNA | mav | fog | pcl | mtDNA | mav | fog | Pcl |
| 1 | HiPt | 1088870 | 582420 | 0.9319 | 0.0135 | 0.0135 | 0.0135 | 1.1919 | 0.0072 | 0.0217 | 0.0361 |
|  | HPD90Lo | 633052 | 278541 | 0.0945 | 0.0135 | 0.0135 | 0.0135 | 0.0939 | 0.0072 | 0.0072 | 0.0072 |
|  | HPD90Hi | 1595320 | 1038217 | 3.6059 | 0.3106 | 0.4997 | 0.4187 | 5.2806 | 0.2962 | 0.6429 | 0.7152 |
| 2 | HiPt | 1139503 | 531767 | 1.2290 | 0.0135 | 0.0135 | 0.0135 | 1.2931 | 0.0072 | 0.0072 | 0.0650 |
|  | HPD90Lo | 633052 | 227909 | 0.1215 | 0.0135 | 0.0135 | 0.0135 | 0.1373 | 0.0072 | 0.0072 | 0.0072 |
|  | HPD90Hi | 1595320 | 987584 | 3.2548 | 0.3106 | 0.4997 | 0.4187 | 5.4395 | 0.2962 | 0.6429 | 0.7007 |
| 3 | HiPt | 1088870 | 531767 | 0.9859 | 0.0135 | 0.0135 | 0.0135 | 1.2208 | 0.0072 | 0.0506 | 0.0650 |
|  | HPD90Lo | 633052 | 227909 | 0.0405 | 0.0135 | 0.0135 | 0.0135 | 0.0795 | 0.0072 | 0.0072 | 0.0072 |
|  | HPD90Hi | 1595320 | 1038217 | 3.5249 | 0.3106 | 0.4997 | 0.4187 | 4.8760 | 0.2962 | 0.6574 | 0.7152 |
| 4 | HiPt | 1139503 | 531767 | 0.9589 | 0.0135 | 0.0135 | 0.0135 | 1.1630 | 0.0072 | 0.0217 | 0.0217 |
|  | HPD90Lo | 683706 | 227909 | 0.2026 | 0.0135 | 0.0135 | 0.0135 | 0.2095 | 0.0072 | 0.0072 | 0.0072 |
|  | HPD90Hi | 1645953 | 987584 | 3.1197 | 0.3106 | 0.4997 | 0.4187 | 5.1216 | 0.3106 | 0.6429 | 0.7152 |
| 5 | HiPt | 1139503 | 582420 | 1.0939 | 0.0135 | 0.0135 | 0.0135 | 1.3653 | 0.0072 | 0.0217 | 0.0795 |
|  | HPD90Lo | 633073 | 227909 | 0.0945 | 0.0135 | 0.0135 | 0.0135 | 0.1228 | 0.0072 | 0.0072 | 0.0072 |
|  | HPD90Hi | 1595320 | 987584 | 3.3628 | 0.3106 | 0.4997 | 0.4187 | 4.9772 | 0.3106 | 0.6429 | 0.7152 |
| 6 | HiPt | 1139503 | 582420 | 1.0399 | 0.0135 | 0.0135 | 0.0135 | 1.0330 | 0.0072 | 0.0217 | 0.0650 |
|  | HPD90Lo | 633052 | 227909 | 0.0135 | 0.0135 | 0.0135 | 0.0135 | 0.1228 | 0.0072 | 0.0072 | 0.0072 |
|  | HPD90Hi | 1595320 | 1038217 | 3.2278 | 0.3106 | 0.4997 | 0.4187 | 4.7749 | 0.2962 | 0.6429 | 0.7007 |
| 7 | HiPt | 1139503 | 531767 | 0.9319 | 0.0135 | 0.0135 | 0.0135 | 0.8452 | 0.0072 | 0.0072 | 0.0506 |
|  | HPD90Lo | 633052 | 227909 | 0.0945 | 0.0135 | 0.0135 | 0.0135 | 0.1373 | 0.0072 | 0.0072 | 0.0072 |
|  | HPD90Hi | 1595320 | 987584 | 3.3898 | 0.3106 | 0.4997 | 0.4187 | 5.6562 | 0.2817 | 0.6429 | 0.7152 |
| 8 | HiPt | 1139503 | 531767 | 1.0399 | 0.0135 | 0.0135 | 0.0135 | 1.0619 | 0.0072 | 0.0361 | 0.0506 |
|  | HPD90Lo | 633052 | 278541 | 0.0135 | 0.0135 | 0.0135 | 0.0135 | 0.2095 | 0.0072 | 0.0072 | 0.0072 |
|  | HPD90Hi | 1595320 | 1038217 | 3.3088 | 0.3106 | 0.4997 | 0.4187 | 4.8327 | 0.2962 | 0.6574 | 0.7152 |
| 9 | HiPt | 1139503 | 531767 | 1.2020 | 0.0135 | 0.0135 | 0.0135 | 1.0185 | 0.0072 | 0.0361 | 0.0795 |
|  | HPD90Lo | 633052 | 227909 | 0.2026 | 0.0135 | 0.0135 | 0.0135 | 0.1517 | 0.0072 | 0.0072 | 0.0072 |
|  | HPD90Hi | 1595320 | 987584 | 2.9306 | 0.3106 | 0.4997 | 0.4187 | 4.8760 | 0.2962 | 0.6574 | 0.7152 |
| 10 | HiPt | 1139503 | 582420 | 0.9319 | 0.0135 | 0.0135 | 0.0135 | 1.1919 | 0.0072 | 0.0506 | 0.0506 |
|  | HPD90Lo | 633052 | 227909 | 0.0675 | 0.0135 | 0.0135 | 0.0135 | 0.1373 | 0.0072 | 0.0072 | 0.0072 |
|  | HPD90Hi | 1595320 | 987584 | 3.0387 | 0.3106 | 0.4997 | 0.4187 | 4.7316 | 0.2962 | 0.6574 | 0.7152 |

HiPt: Highest posterior estimate; HPD90Lo and HP90Hi: Lower and upper bounds of the 90% highest posterior interval of the parameter estimate.

**Table S7. Percentage of *Wolbachia* infected and uninfected individuals for a given fly collection .**

|  | Wolbachia Infected | Uninfected |
| --- | --- | --- |
| *maI* |  |  |
| CS | 78 | 22 |
| MAU | 20 | 80 |
| RED | 100 | 0 |
| RB | 83 | 17 |
| *maII* |  |  |
| MAU | 0 | 100 |
| RED | 0 | 100 |
| *maIII* |  |  |
| MAU | 0 | 100 |
| RED | 0 | 100 |
| RB | 0 | 100 |
| *siIII* |  |  |
| Sita | 50 | 50 |

## Supplementary Figures

**Figure S1. Phylogenetic relationship between *fog* (A), *pcl* (B) and *mav* (C) haplotypes of the *D. melanogaster* subgroup.** Haplotype names carry a prefix of one letter referring to the locus name (F for *fog*, P for *pcl* and M for *mav*) followed by a 3 letter code that refers to the species (MEL, MAU, SIM or SEC, depending on whether they were isolated from *D. melanogaster*, *D. mauritiana, D. simulans* or *D. sechellia* individuals, respectively). Numbers inside the brackets are the frequency of each haplotype in the correspondent species. Bootstrap values, above branches, are calculated based on 100 replicates and indicate the statistical support for the corresponding node.

**A**

**
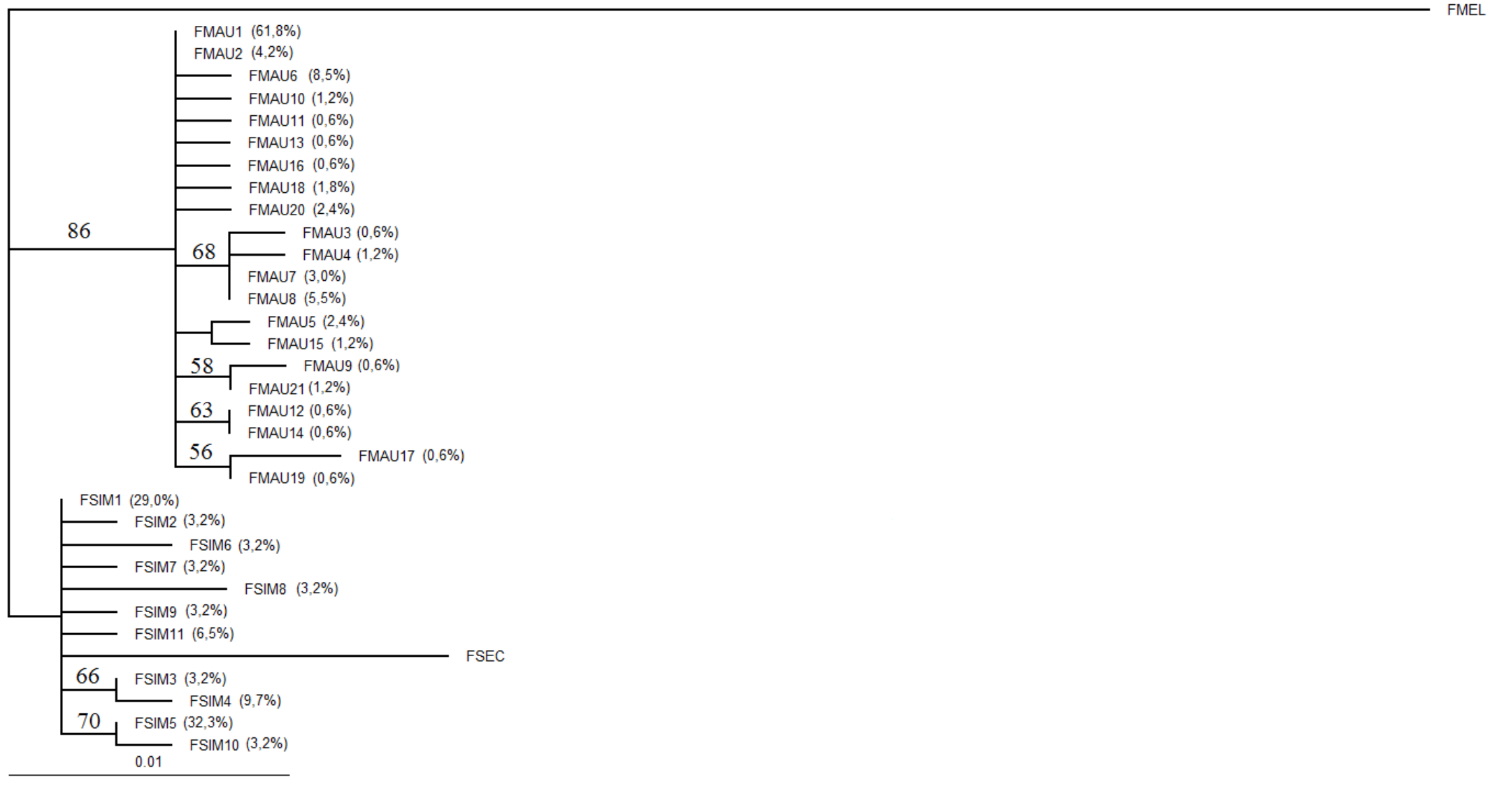
**

B


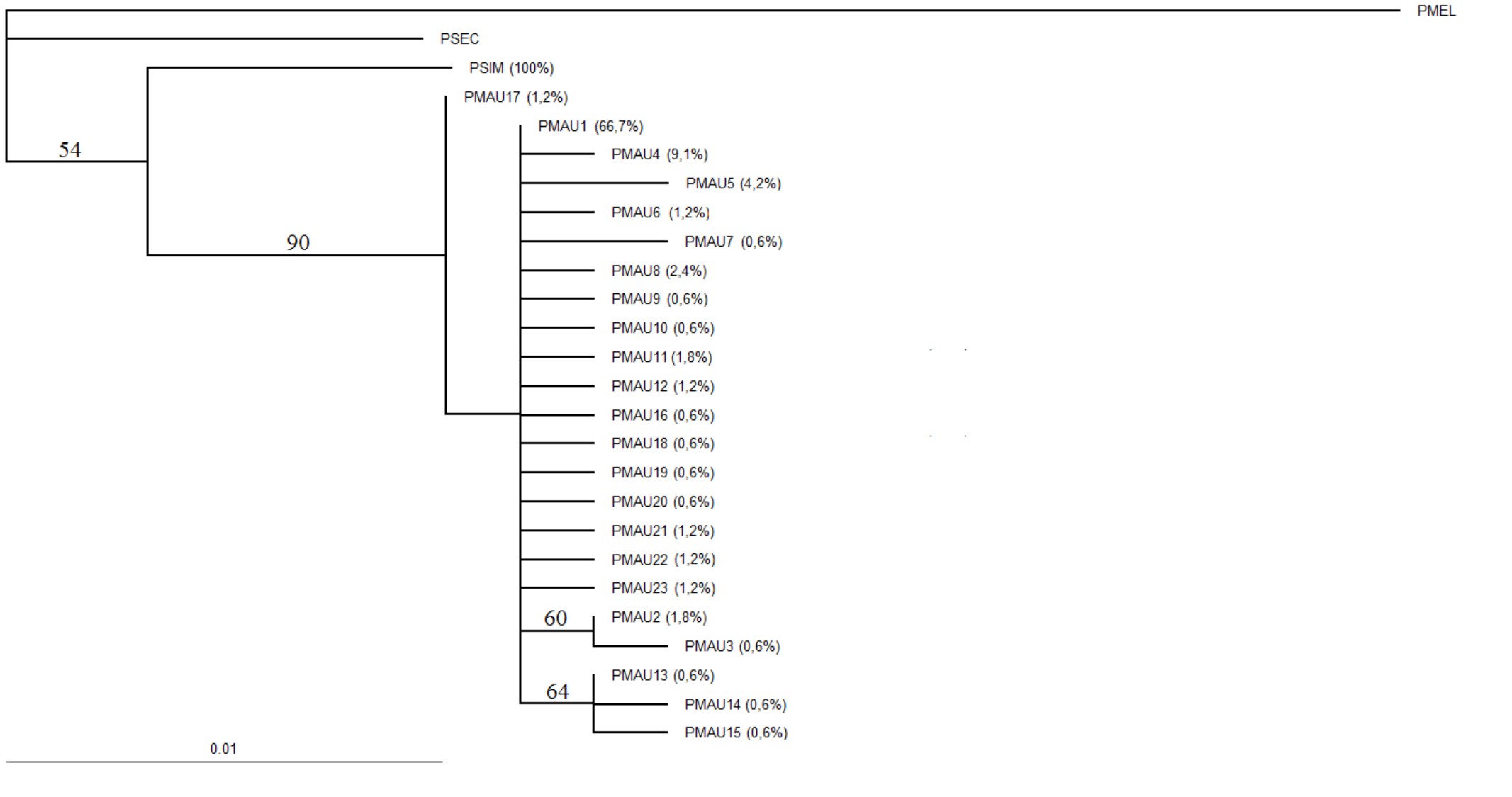


C


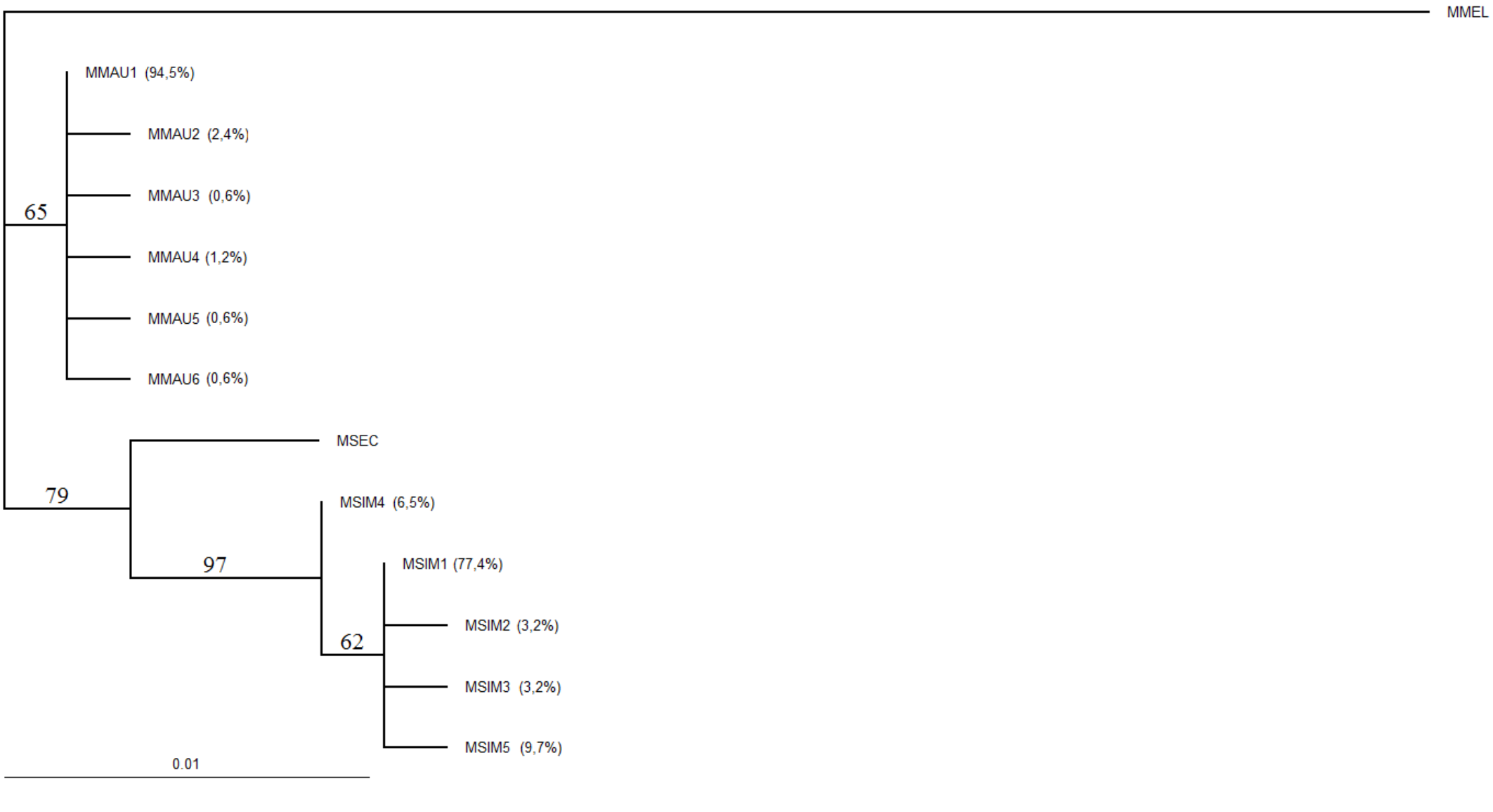


Figure S2. Frequency distribution of the different mitochondrial groups per collection.


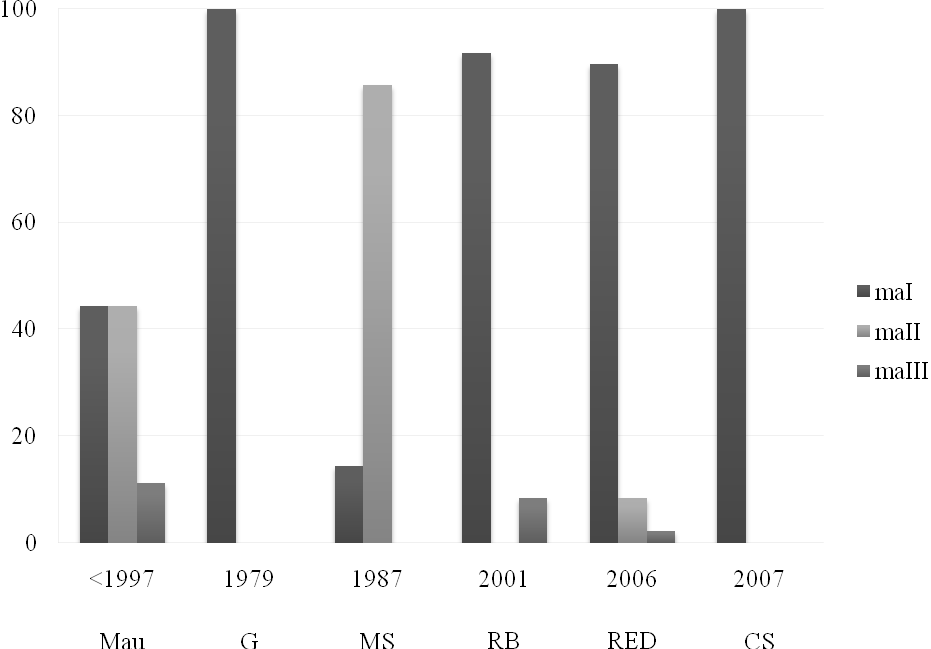

Supplement: Supplementary file 1 [file mec0019-4695-SD1.doc]
